# Targeted Delivery of VEGF-siRNA to Glioblastoma Using Orientation-Controlled Anti-PD-L1 Antibody-Modified Lipid Nanoparticles

**DOI:** 10.3390/pharmaceutics17101298

**Published:** 2025-10-04

**Authors:** Ayaka Matsuo-Tani, Makoto Matsumoto, Takeshi Hiu, Mariko Kamiya, Longjian Geng, Riku Takayama, Yusuke Ushiroda, Naoya Kato, Hikaru Nakamura, Michiharu Yoshida, Hidefumi Mukai, Takayuki Matsuo, Shigeru Kawakami

**Affiliations:** 1 Department of Pharmaceutical Informatics, Graduate School of Biomedical Sciences, Nagasaki University, Nagasaki 852-8588, Japan; heygoo25@live.jp (A.M.-T.); bb55324401@ms.nagasaki-u.ac.jp (M.M.); m82gody@gmail.com (M.K.); l-geng@nagasaki-u.ac.jp (L.G.); bb30120024@ms.nagasaki-u.ac.jp (R.T.); bb30221008@ms.nagasaki-u.ac.jp (Y.U.); tbc_naoya_0922@yahoo.co.jp (N.K.); stradaper@yahoo.co.jp (H.N.); michi511leo@yahoo.co.jp (M.Y.); hmukai@nagasaki-u.ac.jp (H.M.); 2 Department of Neurosurgery, Graduate School of Biomedical Sciences, Nagasaki University, Nagasaki 852-8501, Japan; takayuki@nagasaki-u.ac.jp

**Keywords:** lipid nanoparticles, siRNA, Fc-binding peptide, antibody modification, antibody orientation control, PD-L1, VEGF, targeted drug delivery, glioblastoma

## Abstract

**Background/Objectives**: Glioblastoma (GBM) is an aggressive primary brain tumor with limited therapeutic options despite multimodal treatment. Small interfering RNA (siRNA)-based therapeutics can silence tumor-promoting genes, but achieving efficient and tumor-specific delivery remains challenging. Lipid nanoparticles (LNPs) are promising siRNA carriers; however, conventional antibody conjugation can impair antigen recognition and complicate manufacturing. This study aimed to establish a modular Fc-binding peptide (FcBP)-mediated post-insertion strategy to enable PD-L1-targeted delivery of VEGF-siRNA via LNPs for GBM therapy. **Methods**: Preformed VEGF-siRNA-loaded LNPs were functionalized with FcBP–lipid conjugates, enabling non-covalent anchoring of anti-PD-L1 antibodies through Fc interactions. Particle characteristics were analyzed using dynamic light scattering and encapsulation efficiency assays. Targeted cellular uptake and VEGF gene silencing were evaluated in PD-L1-positive GL261 glioma cells. Anti-tumor efficacy was assessed in a subcutaneous GL261 tumor model following repeated intratumoral administration using tumor volume and bioluminescence imaging as endpoints. **Results**: FcBP post-insertion preserved LNP particle size (125.2 ± 1.3 nm), polydispersity, zeta potential, and siRNA encapsulation efficiency. Anti-PD-L1–FcBP-LNPs significantly enhanced cellular uptake (by ~50-fold) and VEGF silencing in PD-L1-expressing GL261 cells compared to controls. In vivo, targeted LNPs reduced tumor volume by 65% and markedly suppressed bioluminescence signals without inducing weight loss. Final tumor weight was reduced by 63% in the anti-PD-L1–FcBP–LNP group (656.9 ± 125.4 mg) compared to the VEGF-siRNA LNP group (1794.1 ± 103.7 mg). The FcBP-modified LNPs maintained antibody orientation and binding activity, enabling rapid functionalization with targeting antibodies. **Conclusions**: The FcBP-mediated post-insertion strategy enables site-specific, modular antibody functionalization of LNPs without compromising physicochemical integrity or antibody recognition. PD-L1-targeted VEGF-siRNA delivery demonstrated potent, selective anti-tumor effects in GBM murine models. This platform offers a versatile approach for targeted nucleic acid therapeutics and holds translational potential for treating GBM.

## 1. Introduction

Glioblastoma (GBM) is the most malignant primary brain tumor, with an annual incidence of 3.19 to 4.17 cases per 100,000 people worldwide [1]. Despite maximal safe resection, radiotherapy, and temozolomide chemotherapy, median survival rarely exceeds 15 months [1,2,3]. The blood–brain barrier (BBB) and the highly heterogeneous tumor microenvironment severely limit the efficacy of molecularly targeted drugs and immunotherapies [4]. Vascular endothelial growth factor (VEGF), a key driver of angiogenesis, is overexpressed in approximately 90% of GBM cases [5]. Although the anti-VEGF antibody bevacizumab is clinically approved for recurrent GBM, it provides only transient radiographic responses without survival benefit, highlighting the need for more effective VEGF-targeted strategies [6].

RNA interference was first discovered by Fire et al. in 1998, who demonstrated that double-stranded RNA could potently and specifically silence gene expression in Caenorhabditis elegans, establishing the basis for sequence-specific post-transcriptional gene silencing [7]. RNA-based therapeutics, particularly small interfering RNA (siRNA), enable precise silencing of oncogenic or angiogenic targets at the mRNA level and are increasingly being investigated as potential tools for GBM therapy [8,9,10]. 

Among non-viral nucleic acid delivery systems, lipid nanoparticles (LNPs) represent the most clinically validated platform, as evidenced by their successful application in mRNA vaccine development [11,12,13]. However, conventional LNPs lack tumor-targeting specificity, and existing antibody conjugation methods often suffer from random orientation, reduced antigen binding, and manufacturing complexity [14,15]. To overcome these limitations, we previously established the synthesis of the Fc-binding peptide (FcBP)-lipid for targeted delivery of messenger RNA-loaded anti-transferrin antibody-modified LNPs [16].

Here, we developed a modular, GBM-specific targeting platform using the FcBP-lipid for non-covalent post-insertion of antibodies for preformed siRNA-loaded LNP delivery. This approach preserves antibody orientation and recognizing function, enables rapid and scalable manufacturing, and is adaptable to diverse antibodies and RNA cargos. As a proof of concept, we targeted programmed death-ligand 1 (PD-L1), which is expressed in 35–62% of gliomas [17,18], not for immune checkpoint inhibition but as a tumor-selective entry route for VEGF-siRNA delivery. In subcutaneous GBM models, we demonstrate that PD-L1–FcBP-LNPs achieved >60% tumor growth inhibition without systemic toxicity, demonstrating both potent efficacy and clinical translation potential for precision oncology applications.

## 2. Materials and Methods

### 2.1. Materials

Ionizable lipid DLin-MC3-DMA (MC3), DSPC (1,2-distearoyl-*sn*-glycero-3-phosphocholine), cholesterol, and DSG-PEG2000 (1,2-distearoyl-rac-glycero-3-methoxypolyethylene glycol-2000) were obtained from Avanti Polar Lipids (Alabaster, AL, USA). VEGF-targeting siRNA (sense strand sequence: 5′-GGAUCAUUGAAUUAGUACAdTdT-3′) was synthesized by Thermo Fisher Scientific (Waltham, MA, USA) [8,9]. Fc-binding high-functionality and quality lipid (FcBP-HFQ lipid, incorporating an RRGW tetrapeptide Fc-binding motif with an EKGG repeated hydrophilic spacer and a hydrophobic alkyl tail) was synthesized, as described previously [16]. The anti-mouse PD-L1 monoclonal antibody (clone 10F.9G2, rat IgG2b) and the corresponding isotype control antibody (rat IgG2b) were purchased from Bio X Cell (Lebanon, NH, USA). Other reagents were analytical grade and employed as received.

### 2.2. Preparation of VEGF-siRNA LNPs

LNPs were prepared according to our previous reports [19,20] and generated using a microfluidic mixing system (NanoAssemblr^®^ Benchtop, Precision NanoSystems Inc., Vancouver, BC, Canada) [11]. VEGF-siRNA was dissolved in 50 mM citrate buffer (pH 3.0), with a final nucleic acid concentration of 15 mg/mL. A lipid mixture consisting of MC3/DSPC/cholesterol/DSG-PEG2000 at a molar ratio of 50:10:38.5:1.5 was used. The two phases were rapidly mixed at a volume ratio of 3: 1 (siRNA: lipid) with a total flow rate of 4 mL/min (N/P = 12).

### 2.3. Post-Insertion of the FcBP-HFQ Lipid and Antibody Decoration

The FcBP-HFQ lipid was incorporated into preformed LNPs through incubation at 37 °C for 1 h at a final concentration of 1 mol% relative to total lipids. After cooling to room temperature, the anti-PD-L1 antibody or isotype control antibody was added at a lipid to antibody weight ratio of 10:1 (10 *w*/*w*%) and incubated for 1 h. This non-covalent Fc–FcBP interaction oriented the Fab domain outward, enabling selective target recognition.

### 2.4. Physicochemical Characterization

Hydrodynamic diameter, polydispersity index (PDI), and zeta potential were assessed through dynamic light scattering (Zetasizer Nano ZS, Malvern Instruments, Worcestershire, UK) at 25 °C in PBS [11]. siRNA encapsulation efficiency was determined using the Quant-iT™ RiboGreen RNA assay (Invitrogen, Carlsbad, CA, USA). Each experiment was conducted in triplicate, and data are expressed as mean values with standard deviation (SD).

### 2.5. Cell Culture and In Vitro Uptake Assay

The murine glioma cell line GL261 (Cat. No. ACC802) was purchased from Deutsche Sammlung von Mikroorganismen und Zellkulturen GmbH (DSMZ, Braunschweig, Germany). The murine lung carcinoma cell line LLC-1 (Cat. No. RCB0558) was obtained from the RIKEN BioResource Center (RIKEN BRC, Tsukuba, Japan). Murine glioblastoma GL261 cells and LLC-1 were cultured in Dulbecco’s Modified Eagle Medium (DMEM; Gibco, Thermo Fisher Scientific, Waltham, MA, USA) supplemented with 10% fetal bovine serum (FBS; BOVOGEN BIOLOGICALS, Victoria, Australia) and 1% penicillin–streptomycin (Wako Pure Chemical Industries, Ltd., Osaka, Japan) at 37 °C in a humidified 5% CO_2_ atmosphere [18]. Cells were seeded in 24-well plates (5.0 × 10^4^ cells/cm^2^) and incubated with 1.0 μg/mL DiD-labeled siRNA-loaded LNPs for 2 h. After washing twice with PBS, cells were detached with trypsin (0.5% trypsin-5.3 mM EDTA-4Na solution) and analyzed through flow cytometry (BD LSR Fortessa Cell Analyzer, BD Biosciences, San Jose, CA, USA) [21]. Cellular uptake was quantified through mean fluorescence intensity (MFI).

### 2.6. In Vitro Gene Silencing Assay

GL261 cells were seeded at a density of 5 × 10^4^ cells/cm^2^ and cultured for 24 h. Each LNP formulation containing siRNA (final concentration: 1.0 μg/mL) was then added to the cells. After 3 h of incubation, the supernatant was removed using an aspirator, and the medium was replaced. This treatment was repeated every other day. On each indicated day, cell viability was assessed using a WST assay according to the manufacturer’s protocol (Cell Counting Kit-8, FUJIFILM Wako Pure Chemical Corporation, Osaka, Japan). For data analysis, the viability of the PBS-treated control group was set to 100%, and cell viability in the LNP-treated groups was expressed as a percentage of the control to evaluate cell growth inhibition.

### 2.7. In Vivo Tumor Model and Therapeutic Study

Six-week-old male Balb/c nu/nu mice received a subcutaneous implantation of 5 × 10^6^ GL261-luc-GFP cells into the right flank. Mice were randomized into five groups (*n* = 3 per group) when tumor volume reached approximately 100 mm^3^ and intratumorally injected with the following siRNA-LNP formulations (10 μg siRNA/dose) every three days (on days 0, 3, and 6): (1) PBS control, (2) VEGF-siRNA LNP, (3) anti-PD-L1 antibody-modified scramble-siRNA LNP, (4) isotype control antibody-modified VEGF-siRNA LNP, and (5) anti-PD-L1 antibody-modified VEGF-siRNA LNP. Tumor size was monitored daily with a digital caliper, and volume was estimated using the formula 0.5 × length (mm) × width (mm) × height (mm). Tumor weight was recorded at the study’s endpoint. Tumor images were captured using a Canon PC1210 DC7.4V camera. IVIS imaging (IVIS Lumina II, Caliper Life Sciences, Hopkinton, MA, USA) was conducted on days 3, 6, and 9 post-injection to assess biodistribution and tumor-localized signal intensity. Luciferase protein expression in GL261 tumors was monitored using IVIS imaging under 2% isoflurane anesthesia, and the total flux values were quantified from the imaging data. Before imaging, mice were injected with D-luciferin potassium salt (150 mg/kg) intraperitoneally. Imaging was performed 10–15 min after the injection, when the signal was strongest. Balb/c nu/nu mice were purchased from Japan SLC (Hamamatsu, Japan), maintained under specific pathogen-free (SPF) conditions, and had not undergone any previous procedures before tumor inoculation. The animal experiments were approved by the Guidelines for Animal Experimentation of Nagasaki University and the Institutional Animal Care and Use Committee of Nagasaki University (protocol code: 2104011706-2).

### 2.8. Enzyme-Linked Immunosorbent Assay (ELISA)

Tumor tissues administered with each LNP were collected at the endpoint. Tumors were homogenized in lysis buffer and centrifuged at 15,000× *g* for 5 min at 4 °C. The resulting supernatants were collected, and the VEGF protein concentrations were determined using a Mouse VEGF Quantikine ELISA Kit (R&D Systems, Minneapolis, MN, USA; distributed by FUJIFILM Wako Pure Chemical Corporation). The same sample was used for the BCA protein assay following the protocol described for the BCA Protein Assay Kit (Thermo Fisher Scientific, Waltham, MA, USA).

### 2.9. Statistical Analysis

Results are presented as mean ± standard deviation (SD). Statistical significance between two groups was assessed using Student’s *t*-test, whereas one-way ANOVA with Tukey’s post hoc analysis was used for multiple comparisons. A threshold of *p* < 0.05 was applied.

## 3. Results

### 3.1. In Vitro and In Vivo Evaluation of PD-L1-Targeted VEGF-siRNA LNPs

#### 3.1.1. Physicochemical Properties of Antibody-Modified LNPs

The anti-PD-L1 antibody-modified VEGF-siRNA LNPs exhibited a mean particle size of 125.2 ± 1.27 nm (*n* = 3), PDI of 0.18 ± 0.02 (*n* = 3), and a near-neutral zeta potential of –0.74 ± 2.47 mV (*n* = 3). The encapsulation efficiency of VEGF-siRNA exceeded 90%, structural integrity and high loading capacity of the modified LNPs. These results indicate that post-insertion of the FcBP-HFQ lipid and antibody modification did not affect the core physicochemical properties of the LNPs (Table 1).

#### 3.1.2. Targeted Cellular Uptake of LNPs

**Figure 1:**
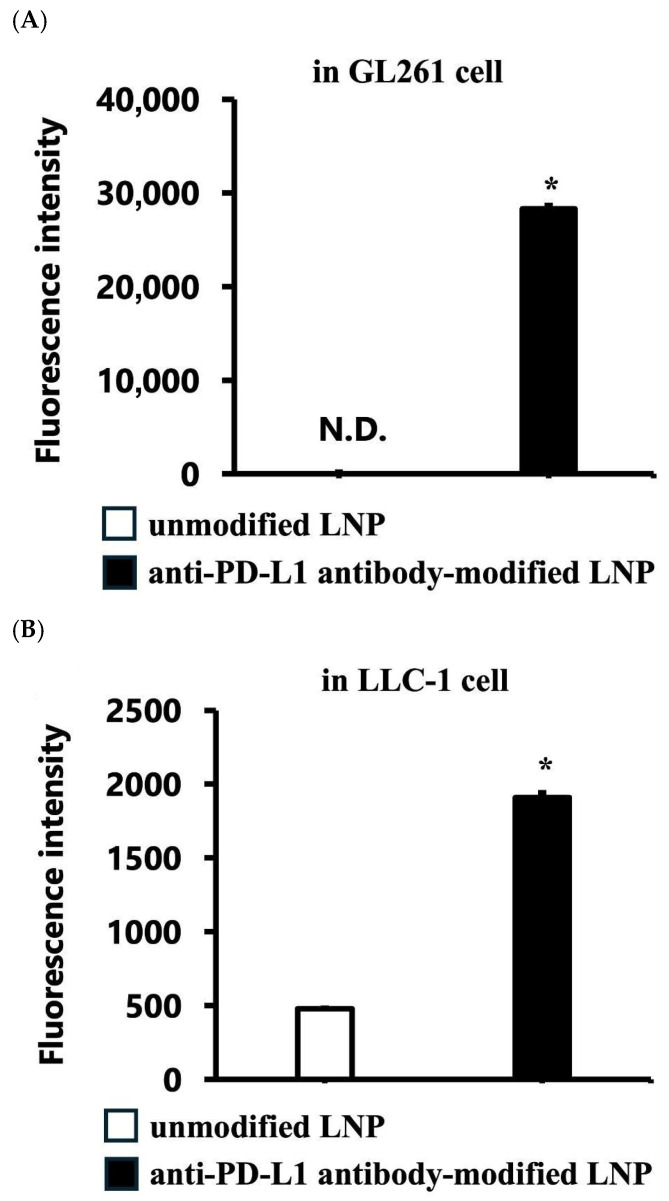
Comparison of the cellular association of unmodified and anti-PD-L1 antibody-modified LNPs. Cells were treated with each DiD-labeled LNP (0.40 μg/mL of siRNA) for 2 h. (**A**) In GL261 glioblastoma cells, anti-PD-L1 antibody-modified LNPs showed approximately 50-fold higher mean fluorescence intensity (MFI) than unmodified and isotype control antibody-modified LNPs (*p* < 0.05). (**B**) In LLC-1 cells, anti-PD-L1 antibody-modified LNPs showed approximately 5-fold higher MFI than unmodified LNPs. Data are presented as the mean ± SD (n = 3). Statistical significance was determined using Student’s *t*-test. * *p* < 0.05. Notes: N.D.: Not detected.

Flow cytometry analysis revealed significantly higher uptake of DiD-labeled siRNA LNPs modified with the anti-PD-L1 antibody in GL261 glioblastoma cells compared with both unmodified and isotype control antibody-modified LNPs (*p* < 0.05). The MFI was ~50-fold higher in the PD-L1-targeted group, demonstrating successful antibody-mediated enhancement of cellular internalization (Figure 1A). In LLC-1 cells, which express fewer PD-L1 receptors than GL261 cells, the MFI was still approximately five-fold higher than in the unmodified group (Figure 1B).

#### 3.1.3. In Vitro Gene Silencing of VEGF

**Figure 2:**
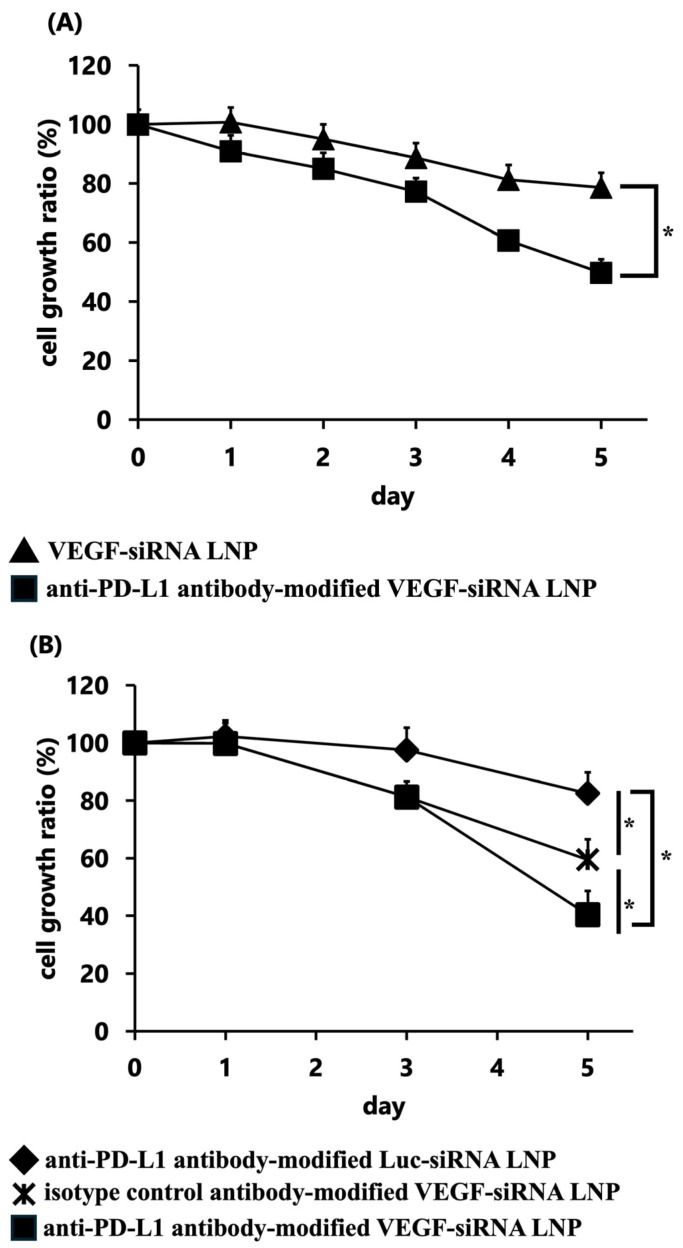
Comparison of the cell growth ratio (%) over 5 days of (**A**) unmodified and anti-PD-L1 antibody-modified LNPs and (**B**) anti-PD-L1 antibody-modified Luc siRNA LNP, anti-PD-L1 antibody-modified VEGF-siRNA LNP, and isotype control antibody-modified VEGF-siRNA LNP. Data are presented as the mean ± SD (*n* = 3). Statistical significance was determined using Student’s *t*-test (**A**) or Tukey’s test (**B**). * *p* < 0.05.

GL261 cells treated with anti-PD-L1 antibody-modified VEGF-siRNA LNPs showed over 50% reduction in the cell growth ratio compared with the untreated control group (*p* < 0.05) (Figure 2A). Moreover, the anti-PD-L1 antibody-modified VEGF-siRNA LNP group showed significantly greater inhibition of proliferation than both the anti-PD-L1 antibody-modified Luc-siRNA LNP group (17.5% knockdown) and the isotype control antibody-modified VEGF-siRNA LNP group (40.5% knockdown), confirming effective and target-specific VEGF gene silencing (Figure 2B).

#### 3.1.4. Inhibition of Tumor Growth Through VEGF Gene Silencing In Vivo

**Figure 3:**
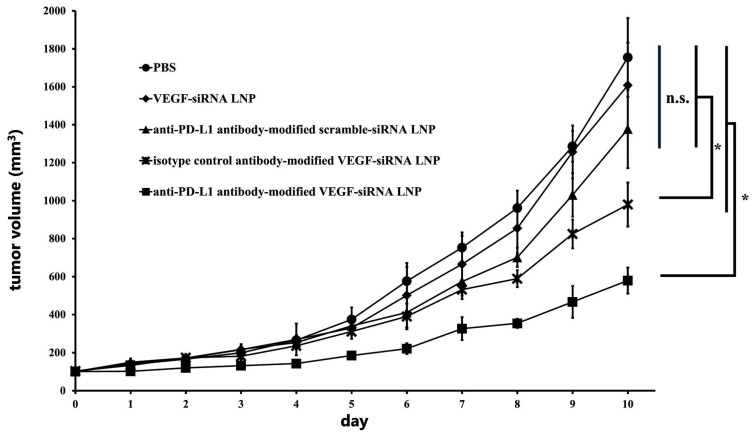
Tumor growth comparison of treatment with PBS, VEGF-siRNA, anti-PD-L1 antibody-modified scramble-siRNA LNP, isotype control antibody-modified VEGF-siRNA LNP, and anti-PD-L1 antibody-modified VEGF-siRNA LNP. Anti-PD-L1 antibody-modified VEGF-siRNA LNPs significantly suppressed tumor growth compared to all other groups and showed smaller tumor volumes throughout the study period (*p* < 0.05). Statistical significance was determined using Tukey’s multiple comparison test. * *p* < 0.05. Notes: n.s.: not significant.

In the GL261-luc-GFP subcutaneous tumor model, anti-PD-L1 antibody-modified VEGF-siRNA LNPs significantly suppressed tumor growth compared to all other groups, maintaining smaller tumor volumes throughout the study period (*p* < 0.05; Figure 3). The mean final tumor volumes (mean ± SD, mm^3^) were PBS, 1754.8 ± 207.6; VEGF-siRNA LNP, 1608.2 ± 224.5; anti-PD-L1 antibody-modified scramble-siRNA LNP, 1377.3 ± 205.8; isotype control antibody-modified VEGF-siRNA LNP, 979.8 ± 115.7; and anti-PD-L1 antibody-modified VEGF-siRNA LNP, 579.2 ± 68.3.

**Figure 4:**
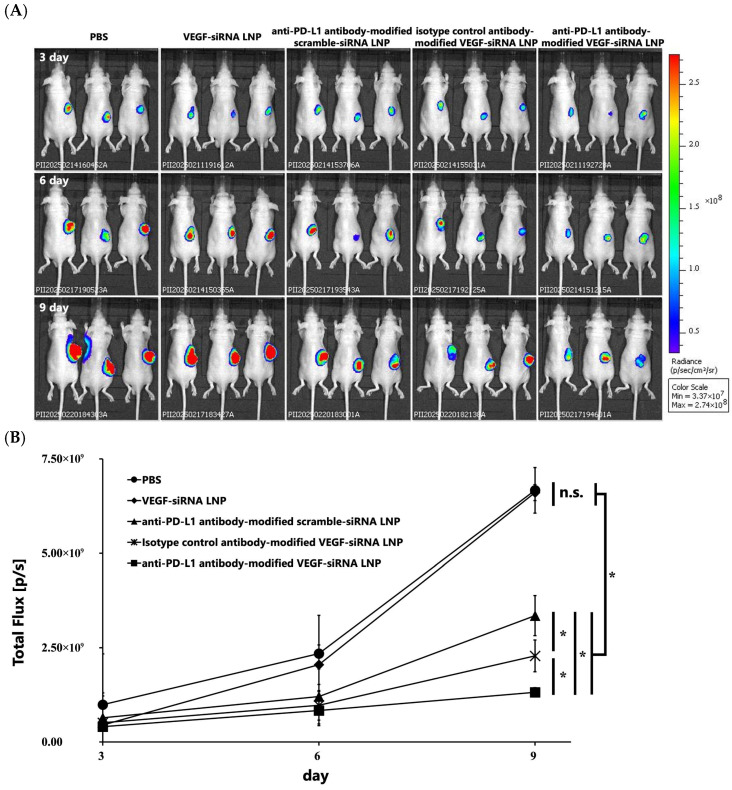
Bioluminescence imaging analysis of tumor growth in mice treated with PBS, VEGF-siRNA LNP, anti-PD-L1 antibody-modified scramble-siRNA LNP, isotype control antibody-modified VEGF-siRNA LNP, and anti-PD-L1 antibody-modified VEGF-siRNA LNP using IVIS. (**A**) IVIS images of mice on days 3, 6, and 9 after intratumoral administration of the indicated formulations. (**B**) Quantitative analysis of luminescent signal intensity (total flux) in tumors treated with PBS, VEGF-siRNA LNP, anti-PD-L1 antibody-modified scramble-siRNA LNP, isotype control antibody-modified VEGF-siRNA LNP, or anti-PD-L1 antibody-modified VEGF-siRNA LNP. Tumors in the anti-PD-L1 antibody-modified VEGF-siRNA LNP group showed significantly lower signals than the other treatment groups, indicating superior tumor growth suppression. Data are presented as the mean ± SD (*n* = 3). Statistical significance was determined using Tukey’s test (B). * *p* < 0.05. Notes: n.s.: not significant.

Furthermore, IVIS imaging conducted on days 3, 6, and 9 post-administration confirmed weaker luminescent signals in tumors treated with anti-PD-L1 antibody-modified LNPs, indicating superior tumor growth inhibition relative to other formulations (Figure 4A,B). On day 9, tumor bioluminescence (total flux; mean ± SD; *n* = 3; units: 10^9^ photons s^−1^) was as follows: PBS, 6.67 ± 2.53; VEGF-siRNA LNP, 6.61 ± 2.05; anti-PD-L1 antibody-modified scramble-siRNA LNP, 3.35 ± 1.08; isotype control antibody-modified VEGF-siRNA LNP, 0.229 ± 0.911; and anti-PD-L1 antibody-modified VEGF-siRNA LNP, 0.132 ± 0.511.

These findings highlight that FcBP-mediated antibody modification enables selective delivery of VEGF-siRNA to PD-L1-positive glioblastoma, resulting in efficient gene silencing and potent suppression of tumor growth in vivo.

#### 3.1.5. Tumor Weight and Size

**Figure 5:**
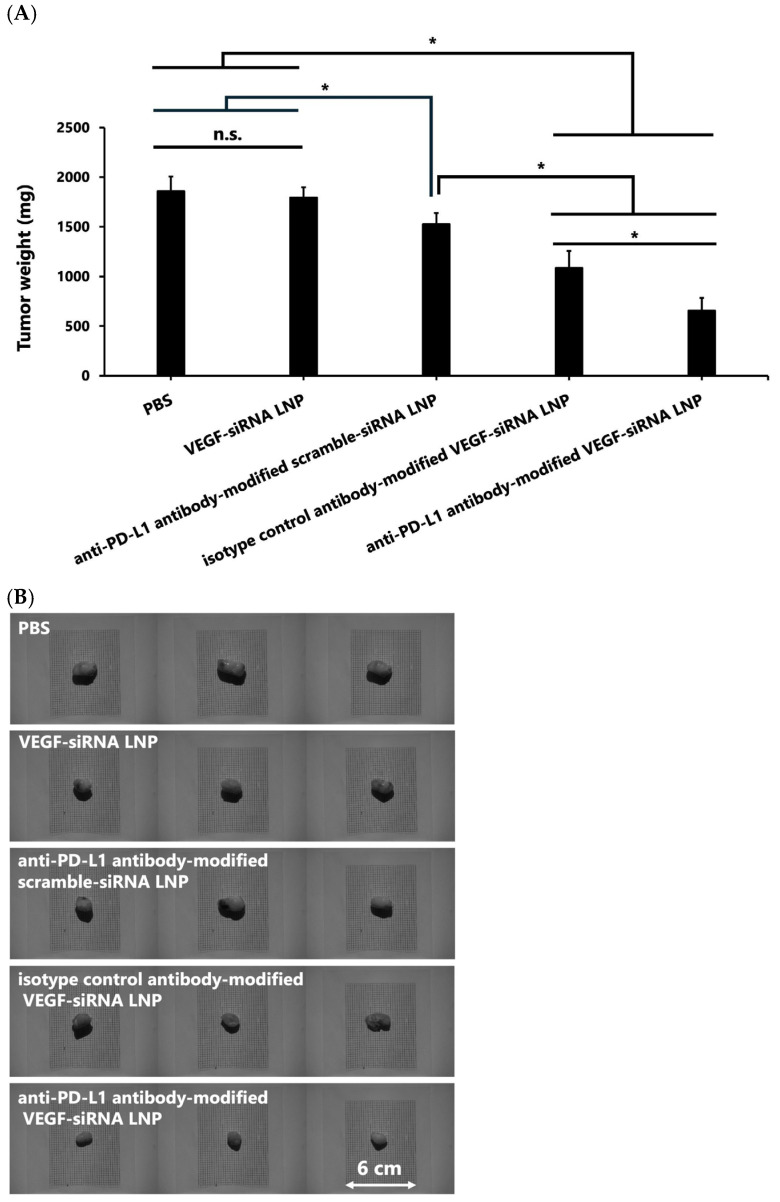
Comparison of tumor weight (**A**) and size (**B**) for each treatment. (**A**) The final tumor weight in the anti-PD-L1 antibody-modified VEGF-siRNA LNP group was significantly lower than that in the other treatment groups. (**B**) Images of tumors show visibly smaller tumors in the anti-PD-L1 antibody-modified VEGF-siRNA LNP group than in the other groups. Statistical significance was assessed using Tukey’s test (A). * *p* < 0.05. Notes: n.s.: not significant.

Final tumor weights were 656.9 ± 125.4 mg (*n* = 3) in the anti-PD-L1 antibody-modified VEGF-siRNA LNP group. In comparison, the weights were 1857.2 ± 148.0 mg (*n* = 3) in the PBS control group, 1794.1 ± 103.7 mg (*n* = 3) in the VEGF-siRNA LNP group, 1523.5 ± 116.5 mg (*n* = 3) in the anti-PD-L1 antibody-modified scramble-siRNA LNP group, and 1085 ± 116.5 mg (*n* = 3) in the isotype control antibody-modified VEGF-siRNA LNP group (Figure 5A). The reduction in tumor weight observed in the anti–PD-L1 antibody–modified VEGF-siRNA LNP group was statistically significant compared to all other groups (*p* < 0.05), representing an approximate 65% reduction relative to the PBS control. The moderate reduction observed in the anti–PD-L1 antibody–modified scramble-siRNA LNP group may be attributed to innate immune activation by duplex RNA, rather than VEGF-specific gene silencing. Additionally, representative tumor images displayed visibly reduced tumor sizes in the anti–PD-L1–VEGF-siRNA LNP group (Figure 5B).

#### 3.1.6. VEGF Concentration in Tumors

**Figure 6:**
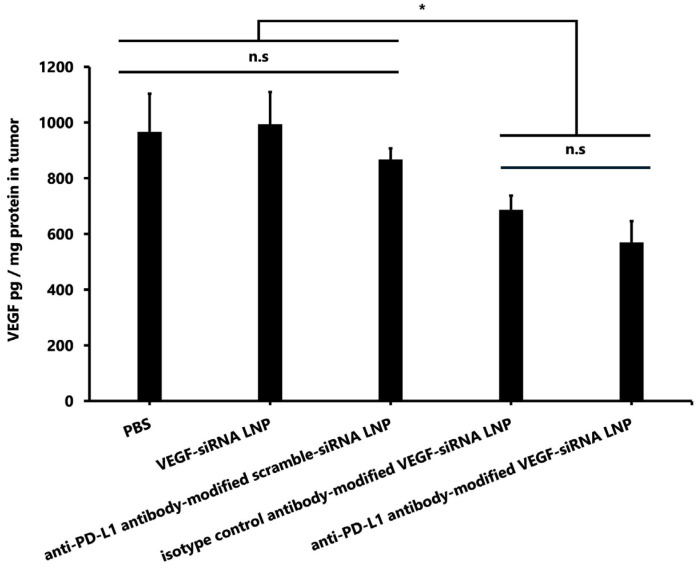
Comparison of VEGF pg/mg protein concentration in tumors for each treatment. Data are presented as the mean ± SD (*n* = 3). VEGF protein levels were significantly lower in the anti-PD-L1 antibody-modified VEGF-siRNA LNP group and the isotype control antibody-modified VEGF-siRNA LNP group than in the PBS, VEGF-siRNA LNP, and anti-PD-L1 antibody-modified scramble-siRNA LNP groups (*p* < 0.05). Statistical significance was assessed using Tukey’s test. * *p* < 0.05. Notes: n.s.: not significant.

Tumoral VEGF protein levels were significantly lower in the anti–PD-L1 antibody-modified VEGF-siRNA LNP group (570.3 ± 75.4 pg/mg protein) compared to the PBS group (966.6 ± 136.4 pg/mg), the unmodified VEGF-siRNA LNP group (993.4 ± 116.0 pg/mg), and the anti-PD-L1 antibody-modified scramble-siRNA LNP group (867.0 ± 40.5 pg/mg) (*p* < 0.05 for all comparisons). However, no significant difference was observed between the anti-PD-L1-modified VEGF-siRNA LNP group and the isotype control antibody-modified VEGF-siRNA LNP group (686.2 ± 50.9 pg/mg) (Figure 6).

## 4. Discussion

Recent advances in RNA therapeutics have increased interest in siRNA for cancer treatment, including GBM [8,9]. However, efficient and tumor-specific delivery of siRNA to solid tumors, such as GBM, remains a major challenge because of poor tumor penetration, rapid systemic clearance, and off-target effects [21,22,23]. LNPs are among the most clinically validated non-viral delivery systems for nucleic acids [24]; however, their inherent lack of tumor-targeting capability limits their therapeutic efficacy without additional surface functionalization [25].

Conventional antibody-mediated LNP targeting strategies often rely on covalent conjugation chemistries, such as maleimide–thiol coupling, which can result in heterogeneous antibody orientation, diminished antigen-binding activity, and added manufacturing complexity [26]. To overcome these limitations, we developed a modular, site-specific targeting platform using FcBP–lipid conjugates. This post-insertion approach enables non-covalent anchoring of antibodies through their Fc region, preserving Fab-mediated antigen recognition and maintaining antibody functionality [16]. In this study, PD-L1 was selected as the surface antigen for antibody-mediated LNP modification, and VEGF was chosen as the siRNA target. The aim was not to evaluate the intrinsic anti-tumor effects of PD-L1 inhibition but rather to exploit PD-L1 expression as a tumor-selective delivery route for siRNA therapeutics. Therefore, we established a subcutaneous tumor model in nude mice with GL261 cells to avoid T-cell-mediated immune responses. PD-L1 was selected as the targeting receptor because 35–62% of glioma patients exhibit PD-L1 expression ≥5% [18]. VEGF-siRNA was chosen because VEGF is overexpressed in approximately 90% of GBM cases [5] and plays a critical role in tumor angiogenesis.

Our results demonstrate that FcBP–lipid can be efficiently incorporated into preformed VEGF-siRNA LNPs without altering key physicochemical parameters, including particle size, PDI, zeta potential, and encapsulation efficiency, which is consistent with previous reports using EKGG- and RRGW-based FcBP conjugates [16]. Decoration with anti-PD-L1 antibodies significantly enhanced cellular uptake (Figure 1) and VEGF gene silencing in PD-L1-positive GL261 glioma cells compared with isotype controls, confirming target specificity. In vitro, anti-PD-L1–FcBP–LNPs suppressed VEGF mRNA expression by >70% and inhibited cell proliferation (Figure 2). In vivo, repeated intratumoral administration in a subcutaneous GL261 model markedly suppressed tumor growth (Figure 3), reduced bioluminescent signals (Figure 4), and decreased tumor volume by 65% compared to controls (Figure 5) without affecting body weight. This tumor-suppressive effect was statistically significant, suggesting that FcBP–LNPs achieved functional VEGF knockdown with minimal off-target toxicity.

The reduction in bioluminescent signals over time (Figure 4A,B) provides additional evidence of tumor suppression and delivery specificity. Serial IVIS imaging on days 3, 6, and 9 revealed a progressive decrease in luminescent intensity in tumors treated with PD-L1–FcBP–LNPs, indicating sustained inhibition of tumor progression. In contrast, tumors treated with control LNPs or isotype antibody-modified LNPs showed no significant signal reduction, highlighting the importance of antigen-specific targeting. Moreover, the >60% reduction in tumor volume (Figure 5) strongly correlates with the observed VEGF protein suppression (Figure 6), supporting a mechanistic link between VEGF knockdown and tumor regression. These findings are consistent with previous reports demonstrating that VEGF-driven angiogenesis plays a central role in GBM growth. Most previous studies on PD-L1 in GBM have focused on immune checkpoint inhibition by blocking PD-L1/PD-1 signaling, rather than exploiting PD-L1 as a delivery target [18,27,28]. Our findings position PD-L1 as a promising entry point for LNP-based therapeutics. Anti-VEGF therapy with bevacizumab has not improved the overall survival of GBM patients [6]. In contrast, our results indicate that siRNA-mediated VEGF silencing, especially with tumor-targeted delivery, can exert potent anti-tumor effects. A plausible explanation is that antibody-based VEGF inhibition may trigger compensatory angiogenic pathways, whereas gene-level knockdown suppresses VEGF production more directly. This activity also raises the possibility of combining VEGF-siRNA with inhibitors targeting alternative angiogenesis pathways to prevent vascular escape mechanisms [5].

In addition, anti-PD-L1 antibody-modified VEGF-siRNA LNPs may confer dual functionality by acting as immune checkpoint inhibitors in a normal host immune system, enabling synergistic anti-tumor responses. The modular FcBP-based approach allows for rapid customization with various antibodies and nucleic acid cargos, supporting the development of personalized nanomedicine. The RRGW peptide used herein binds the Fc region with high affinity (*K*_D_ ≈ 0.5 nM) [15], ensuring stable antibody anchoring even in the presence of endogenous IgG, which is a critical feature for clinical translation.

Despite these promising findings, this study has limitations. The subcutaneous tumor model does not replicate the BBB or the complex GBM microenvironment. Although IVIS imaging confirmed tumor-specific accumulation, validation in orthotopic glioma models will be essential. In this regard, recent work by Tang et al. [29] has demonstrated BBB-permeable PD-L1-targeted siRNA–LNPs, supporting the feasibility of antibody-guided delivery to brain tumors. Incorporating non-invasive BBB disruption strategies, such as focused ultrasound and microbubbles, may further enhance brain penetration [4,20]. Additionally, intratumoral administration, while effective in this study, is an inherently localized therapy with limited systemic applicability. However, localized gene therapy approaches have been investigated for GBM [30], suggesting that nucleic-acid-based local delivery is a feasible regulatory pathway. Further limitations include the lack of long-term safety data, immune-related toxicity assessment, and evaluation of repeated dosing, which will require further preclinical studies.

From a clinical perspective, the FcBP–LNP platform has several important implications. GBM remains highly refractory to existing treatments, and the proposed system provides a flexible framework for selective siRNA delivery, potentially reducing off-target effects and improving therapeutic efficacy. Beyond siRNA, this platform may accommodate other nucleic acid cargos, including mRNA, miRNA, or CRISPR/Cas9 components [31]. For example, IL-12 mRNA delivery to enhance immune activation or gene editing targeting GBM oncogenes may significantly broaden the therapeutic scope of this platform [32].

## 5. Conclusions

In conclusion, we have demonstrated a versatile and reproducible FcBP-mediated LNP system for VEGF-siRNA delivery via anti-PD-L1 antibody modification. This strategy preserves antibody activity and maintains nanoparticle stability, thereby achieving potent and specific tumor suppression through localized administration. Our findings lay the groundwork for further development of FcBP–LNP platforms as clinically translatable nanomedicines for GBM treatment.

## Figures and Tables

**Table 1:** Core physicochemical properties of modified LNPs.

	Size (nm)	PDI	Zeta Potential (mV)	EE (%)
VEGF-siRNA LNP	109.93 ± 1.82	0.05 ± 0.01	–5.09 ± 3.55	94.23 ± 0.93
Anti-PD-L1 antibody-modified scramble-siRNA LNP	118.97 ± 1.27	0.18 ± 0.04	–3.79 ± 1.47	93.77 ± 1.35
Isotype control antibody- modified VEGF-siRNA LNP	119.2 ± 1.82	0.13 ± 0.01	–2.97 ± 0.85	93.53 ± 0.93
Anti-PD-L1 antibody-modified VEGF-siRNA LNP	125.23 ± 1.27	0.18 ± 0.02	–0.74 ± 2.47	93.37 ± 1.21

Notes: PDI: polydispersity index; EE: encapsulation efficiency. The results are presented as mean ± SD (*n* = 3).

## Data Availability

The original contributions presented in this study are included in the article. Further inquiries can be directed to the corresponding authors.

## Abbreviations

The following abbreviations are used in this manuscript:

GBM	Glioblastoma
siRNA	Small interfering RNA
LNPs	Lipid nanoparticles
FcBP	Fc-binding peptide
BBB	Blood–brain barrier
VEGF	Vascular endothelial growth factor
PD-L1	Programmed death-ligand 1
MC3	DLin-MC3-DMA
DSPC	1,2-distearoyl-*sn*-glycero-3-phosphocholine
DSG-PEG2000	1,2-distearoyl-rac-glycero-3-methoxypolyethylene glycol-2000
FcBP-HFQ lipid	Fc-binding high-functionality and quality lipid
PDI	Polydispersity index
SD	Standard deviation
